# Safety and anti-tumor effects of vismodegib in patients with refractory advanced gastric cancer: A single-arm, phase-II trial

**DOI:** 10.7150/jca.67050

**Published:** 2022-01-09

**Authors:** Ryul Kim, Jun Ho Ji, Jung Hoon Kim, Jung Yong Hong, Ho-Yeong Lim, Won Ki Kang, Jeeyun Lee, Seung Tae Kim

**Affiliations:** 1 Division of Hematology-Oncology, Department of Medicine, Samsung Medical Center, Sungkyunkwan University School of Medicine, Seoul, Korea.; 2 Department of Internal Medicine, Samsung Changwon Hospital, Sungkyunkwan University School of Medicine, Changwon, Korea.; 3 Division of Hematology-Oncology, Department of Internal Medicine, Gyeongsang National University Hospital, Jinju, Korea.

**Keywords:** Advanced gastric cancer, Hedgehog pathway, Phase II clinical trial, SMO, Vismodegib

## Abstract

This phase-II study (ClinicalTrials.gov identifier: NCT03052478) aimed to evaluate the efficacy and safety of vismodegib, an inhibitor targeting the Hedgehog signaling pathway, in patients with refractory advanced gastric cancer. Patients with refractory advanced gastric cancer, whose disease had progressed after undergoing standard therapies, were enrolled in this phase-II trial of vismodegib. Vismodegib (150 mg) was administered orally once a day for a 21-day cycle. The primary endpoint was objective response rate, and the secondary endpoints were overall survival and safety profile. Tumor biopsies were obtained before vismodegib treatment. We conducted whole-exome and transcriptome sequencing to analyze biomarkers. Twenty-three patients were enrolled in this study. Among 19 patients who were eligible for response evaluation, only one showed stable disease, yielding a disease control rate of 5.3%. Median overall survival was 74 days (95% confidence interval, 74-151 days). Treatment-related adverse events of any grade were reported in seven patients (31.8%), and most were grade 1 or 2. Whole transcriptome data showed that the Hedgehog signaling pathway was not enriched in patient samples. This is the first clinical trial demonstrating the clinical activity and safety of vismodegib monotherapy in refractory advanced gastric cancer patients. Further well-designed clinical trials should be conducted to select advanced gastric cancer patients who are likely to benefit from vismodegib.

## Introduction

Gastric cancer (GC) is the fifth most common malignancy and the third leading cause of cancer-related death worldwide 1, 2. Despite advances in GC treatment, the prognosis of advanced gastric cancer (AGC) remains extremely poor, with median overall survival of ~1 year. Patients with a history of treated AGC have an even worse prognosis 3. Although several novel agents have been shown to increase survival as the third or later treatment line, there are few treatment options for patients with AGC who have progressed to second- or third-line treatment, underscoring the need for effective therapies with acceptable safety profiles 4.

Recent molecular and genetic studies found several cancer types with aberrantly activated Hedgehog (Hh) signaling pathway, which plays an important role in inflammation and carcinogenesis 5. Therefore, targeting Hh signaling has generated substantial interest. Two small-molecule inhibitors (GDC-0449 [vismodegib] and LDE225) have shown clinical efficacy in basal cell carcinoma and medulloblastoma and have received FDA approval 6, 7. GDC-0449 antagonizes Hh signaling by binding to the extracellular domain of SMO (Smoothened, a frizzled-class receptor), a transmembrane receptor protein that delivers signals from Hedgehog ligands to cells. About 1.44% of GC patients have altered SMO, which leads to its constitutive activation 8, and Hh inhibitors suppressed tumor proliferation and invasion of gastric cancer in preclinical studies 9-11.

Here, we designed a phase-II study of vismodegib in patients with refractory AGC who experienced failure of standard chemotherapies. We aimed to evaluate the efficacy and safety of vismodegib as a salvage therapy in refractory AGC patients. Additionally, we explored the genomic characteristics related to the anti-tumor activity of vismodegib.

## Materials and Methods

### Study design and patients

This trial was a multicenter, open-label, single-arm, phase-II study conducted at three centers in the Republic of Korea (ClinicalTrials.gov identifier: NCT03052478). Eligible patients were required to meet the following criteria: (1) at least 20 years old, (2) refractory AGC (including gastroesophageal junction adenocarcinoma) that progressed during or after first- or second-line therapy, (3) adequate organ function per protocol, (4) at least one measurable lesion according to the Response Evaluation Criteria in Solid Tumors (version 1.1) 12, and (5) Eastern Cooperative Oncology Group (ECOG) performance status of 0 or 1. All patients were naive to prior treatment with Hedgehog pathway inhibitor and provided written informed consent before enrollment.

Vismodegib (150 mg) was administered orally once a day for a 21-day cycle. The dosing schedule was chosen based on a previous trial 13. Patients received vismodegib until they experienced disease progression, unacceptable toxic effects, or when the study was discontinued. Dose interruption up to four weeks was allowed for patients to recover from toxic effects. Toxicities were graded based on the National Cancer Institute Common Terminology Criteria for Adverse Events 4.0.

The primary endpoint was objective response rate (ORR), which was assessed by independent review using the RECIST guidelines, version 1.1 12. The secondary endpoints were overall survival (OS) and safety profile. The trial protocol was approved by the Institutional Review Board of Samsung Medical Center (Seoul, Korea; IRB No. 2016-08-130) and was conducted in accordance with the Declaration of Helsinki and the Guidelines for Good Clinical Practice.

### Tumor sample collection

Pre-treatment tumor tissue was obtained between 42 days and 1 day before initiation of study treatment. After quality assessment of the biopsy samples, we extracted tumor DNA and RNA from freshly obtained tumor and blood tissues for whole-exome and transcriptome sequencing (**Supplementary Methods**). Indexed libraries were submitted to an Illumina HiSeq2500 (Illumina, San Diego, CA, USA), and paired-end (2x100bp) sequencing was performed by Macrogen Inc. (Republic of Korea).

### Variant calling and filtering of whole-exome and transcriptome sequences

Sequenced reads were mapped to the human reference genome (GRCh37) using the BWA-MEM algorithm 14. The duplicated reads were removed by Picard (available at http://broadinstitute.github.io/picard), and indel realignment and base quality score recalibration were performed by GATK 15. To establish a highly sensitive set of somatic single nucleotide variants (SNVs) and short indels, we collected the unions of variant calls from Mutect2 and Varscan2 16, 17. Variants called by both of the tools were included for future analysis. To establish high-confidence somatic variant sets, we applied additional filtering processes. Additionally, somatic copy-number alterations (CNAs; i.e., mutational signatures) were analyzed using an in-house bioinformatics pipeline (**Supplementary Methods**).

We aligned whole transcriptome sequences using the STAR algorithm 18 and processed them according to the RNA-sequencing (RNA-seq) pipeline recommended by ENCODE (https://www.encodeproject.org/pipelines/). The Gene Set Variant Analysis (GSVA) and Gene Set Enrichment Analysis (GSEA) algorithms were used to explore the whole transcriptome dataset 19, 20.

### Statistical analysis

Antitumor activity was assessed in all patients who received at least one vismodegib dose and had at least one post-baseline scan. Safety was evaluated in all patients who received at least one vismodegib dose. An estimated sample size of 26 was necessary to accept the hypothesis that the true DCR was 30% with 80% power and to reject the hypothesis that the response rate was less than 10%, with a one-sided alpha of 10%. The OS measured from the start of treatment to the date of death from any cause was estimated using the Kaplan-Meier method. All statistical tests were carried out using R version 3.6.0 (http://www.r-project.org).

## Results

### Clinicopathological characteristics of the study participants

From Feb 20, 2017 to May 20, 2019, 23 patients were enrolled in this study. Their clinicopathological characteristics are summarized in **Table 1**. The median age was 61 (range: 33-83) years, and men constituted ~70% of the patients. All the patients had ECOG performance status of 1. Primary tumors were mostly located in the stomach body (n=13, 56.5%) or antrum (n=8, 34.8%). Four patients (17.4%) had HER2-positive AGC (defined as immunohistochemistry 3+ or 2+ with *HER2*:CEP17 fluorescence *in-situ* hybridization ratio ≥2.0). Only one patient was positive for Epstein-Barr virus. Most patients (n=19, 82.6%) received vismodegib as a third or later line of treatment. One patient (ID3) died before starting vismodegib treatment, and three patients (ID7, ID8, and ID12) were lost to follow-up before response evaluation.

### Vismodegib anti-tumor activity

**Figure 1:**
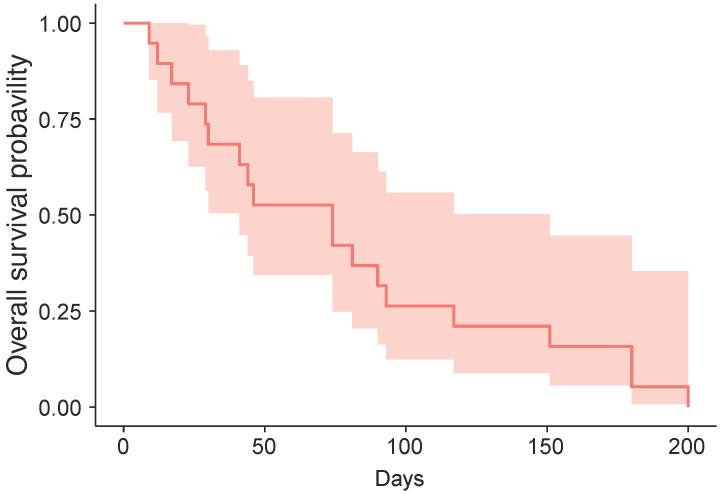
Kaplan-Meier estimate of overall-survival.

The cutoff date for treatment outcome analysis was July 17, 2019, at which time response evaluations were available for 19 patients (82.6%) (**Table 2**). We identified one case (ID6) of stable disease, yielding a DCR of 5.3%; the patient received five cycles of vismodegib and was alive at the cutoff date. At the date cutoff, 22 of 23 patients had died, and the median OS was 74 days (95% confidence interval: 74-151 days; **Figure 1**).

### Genetic features of enrolled patients

**Figure 2:**
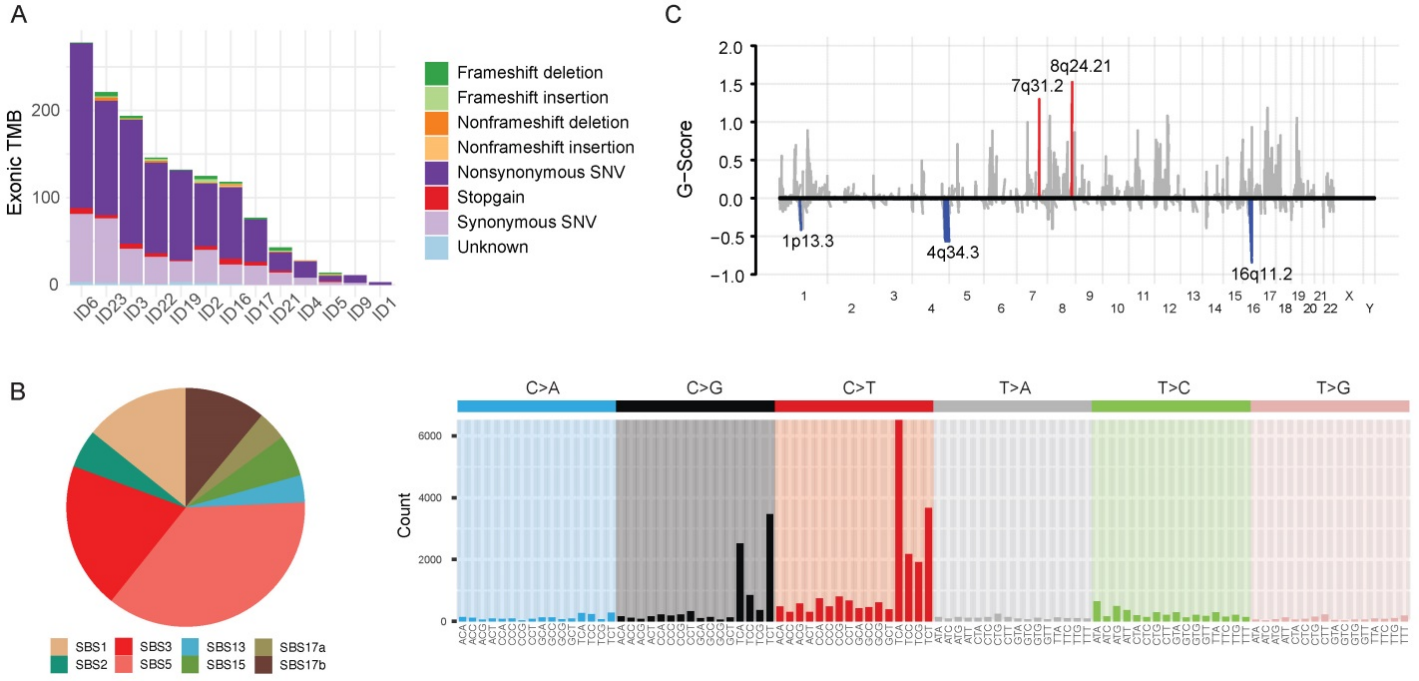
** Genomic characteristics of 13 advanced gastric cancer samples. A.** Barplot illustrating each patient's exonic tumor mutational burden. **B.** Signatures of exonic somatic single-base substitutions (SBSs) delineated by COSMIC signatures. **C.** Genomic plot with segments highlighting significant amplification (red, above the horizontal line) and deletion (blue, below the horizontal line) regions. The G-score was assigned by the Genomic Identification of Significant Targets in Cancer algorithm (GISTIC; see Supplementary Methods) according to the amplitude of aberration and frequency of occurrence across samples. False Discovery Rate q-values were calculated for the aberrant regions, and regions with a q-value <0.10 were considered significant. **Abbreviations:** TMB, tumor mutational burden; SNV, single-nucleotide variation; SBS, single-base substitution.

Tumor and matched-blood samples were available in 13 patients. We analyzed whole-exome sequences (WES) of those samples in a unified pipeline (mean sequencing coverage of ~200x for tumor and matched blood samples). We found high-confidence somatic mutations, including 27,850 base substitutions and 1,489 indels (**Figure 2A**). The samples displayed a variable number of somatic mutations, with a mean of 439 (range 2-3196), which is slightly fewer than that found by The Cancer Genome Atlas (**Supplementary Figure 1A**). Most of the single-nucleotide substitutions were C:G<T:A (43.8%, interquartile range [IQR]: 33.3%-50.0%) (**Supplementary Figure 2**). The mutational spectra suggest that previously defined mutational signatures of endogenous processes, such as SBS1 (5-methylcytosine deamination) and SBS5 (unknown etiology), as well as SBS3 (defective homologous recombination-based DNA damage repair) and SBS17b (unknown etiology) were predominantly responsible for somatic single-nucleotide variations (**Figure 2B**; see also the COSMIC database https://cancer.sanger.ac.uk/cosmic/signatures for the latest mutational signatures).

We analyzed recurrent somatic copy number alterations (CNAs) using WES data (**Figure 2C**). The Genomic Identification of Significant Targets in Cancer (GISTIC) algorithm identified eight amplified and six deleted recurrent focal somatic CNAs. These focal regions were reported previously to be altered in AGC, although some had not been previously implicated (**Supplementary Table 2**).

### Activity of the Hedgehog signaling pathway in whole-transcriptome sequences

**Figure 3:**
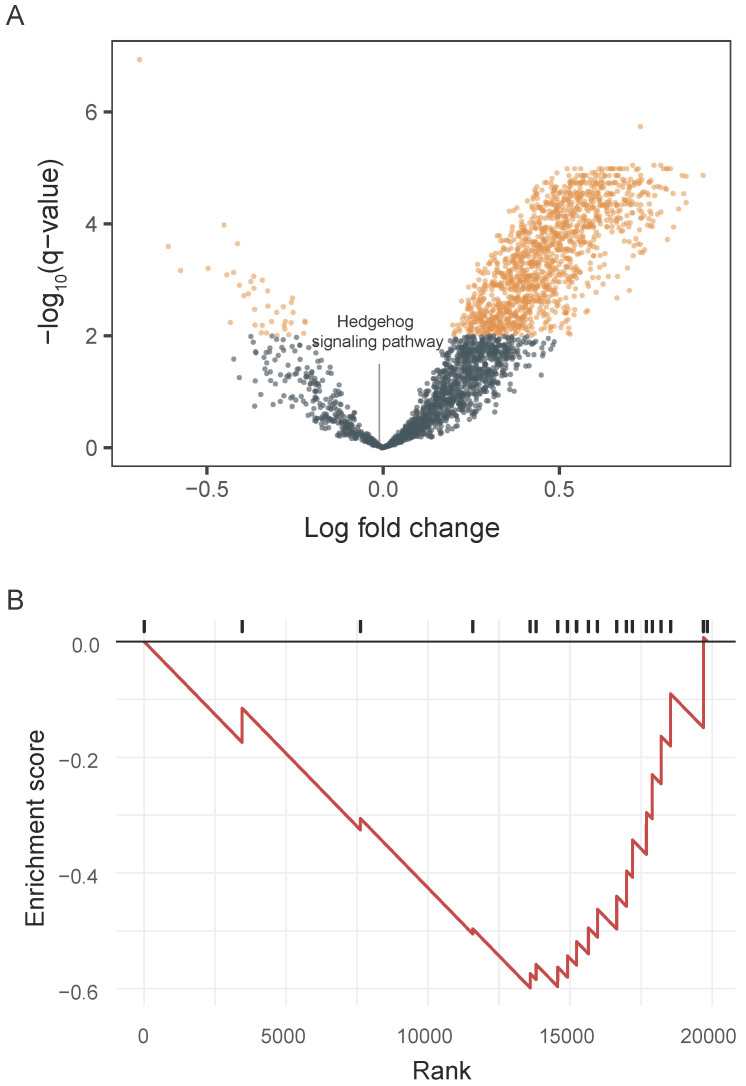
** Pathway enrichment in advanced gastric cancer samples. A.** Volcano plot showing differentially activated pathways between tumor samples and matched normal blood samples. We performed Gene Set Variation Analysis (GSVA) to screen the most significantly activated or suppressed signaling pathways. **B.** Gene Set Enrichment Analysis plot showing downregulation of the SMO activation pathway.

We integrated tumor and matched-normal RNA-seq from 11 patients to characterize Hh signaling pathway activity (**Figure 3**). We performed GSVA to screen the most significantly activated or suppressed signaling pathways in the enrolled patients. Overall, 1369 differentially activated pathways were identified: 1331 activated gene sets and 38 suppressed gene sets (p-value <0.05; **Figure 3A**). The Hh signaling pathway was not enriched in patient samples from this study, explaning the poor DCR observed in this study. Furthermore, GSEA revealed that genes involved in SMO activation also were not enriched in patient samples (**Figure 3B**).

### Safety

With a median of two treatment cycles (range: 1-5 cycles), treatment-related adverse events (TRAEs) of any grade were reported in seven patients (31.8%), and most were grade 1 or 2 (**Table 3**). Grade 3 TRAEs occurred in two patients (9.0%), and a Grade 4 TRAE was reported in one patient (4.5%) with hyperbilirubinemia. None of the patients died or required does interruption as a result of TRAEs.

## Discussion

In this phase II trial, we demonstrated the efficacy and safety of GDC-0449 (vismodegib), the first Hh signaling pathway-targeting agent, in refractory AGC patients. Stable disease was observed in only one patient (ID6), resulting in a disease control rate of 5.3%, and the median overall survival was 2.4 months, with a modest safety profile. Additionally, the genes involved in the Hh signaling pathway were not enriched in patient samples.

Mutations in SMO that lead to constitutive activation play a role in carcinogenesis of various cancers 21. The FDA has approved vismodegib for treatment of metastases and locally advanced basal cell carcinoma 6, 7. Based on the activated Hh signaling pathway in a subset of AGC patients and preclinical activity of vismodegib on gastric cancer cell lines 9-11, several clinical trials have studied the efficacy of vismodegib in AGC. However, no positive effects have been observed 22.

Given the complexity of Hh signaling and the heterogeneity of AGC, the precise mechanisms of Hh signaling need to be studied further for the validation of therapeutic targets and ideal biomarkers. Our analyses indicate that patients enrolled in this study did not have an enriched Hh signaling pathway. Further blood and tissue biomarker analyses should be conducted to determine if there is a subset of patients who derive benefit from vismodegib. Indeed, a subset of AGC patients with CD44 overexpression, a gastric cancer stem-cell biomarker, had better overall survival when treated with vismodegib in combination with cytotoxic chemotherapy 23.

This study has several limitations that limit the generalizability of our results. First, the sample size was very small, and the enrolled patients were not enriched with a biomarker for the Hh signaling pathway. Second, only a subset of patients was eligible for whole-exome and transcriptome sequencing. Therefore, the genomic landscape of the patients could not be described accurately.

To our knowledge, this is the first clinical trial to demonstrate the clinical activity and safety of vismodegib monotherapy in refractory AGC patients. Although the efficacy of vismodegib was limited, the clinical utility of Hh inhibitors for GC should be evaluated further with well-designed, biomarker-driven, clinical trials, for which our data can serve as the basis.

## Supplementary Material

Supplementary figures.

## Figures and Tables

**Table 1:** Clinicopathological characteristics of participants.

ID	Age	Sex	Location	HER2 IHC	EBV	Study line
ID1	83	M	Antrum	3+		3rd
ID2	58	M	Angle	3+		4th
ID3	80	M	Antrum	3+		
ID4	61	F	Body	0		3rd
ID5	61	F	Body	0	-	3rd
ID6	62	F	Body	0		5th
ID7	33	M	Body	0	-	3rd
ID8	65	M	Antrum	0	-	4th
ID9	56	M	Antrum	0		3rd
ID10	57	M	Antrum	0		4th
ID11	63	F	Antrum	2+	-	3rd
ID12	62	M	Body	2+	-	2nd
ID13	38	M	Body	0	-	3rd
ID14	60	M	Body	0	-	6th
ID15	67	M	Cardia	0	+	2nd
ID16	77	M	Antrum	0	-	5th
ID17	71	M	Body	0	-	5th
ID18	33	F	Body	3+	-	3rd
ID19	40	M	Body	0	-	6th
ID20	74	F	Antrum	0	-	4th
ID21	54	F	Body	0	-	2nd
ID22	66	M	Body	0	-	6th
ID23	60	M	Body	0	-	5th

Abbreviations: IHC, immunohistochemistry; EBV, Epstein-Barr virus; M, male; F, female.

**Table 2:** The treatment response of vismodegib.

ID	Cycles	BOR
ID1	1	PD
ID2	1	PD
ID3		
ID4	4	PD
ID5	1	PD
ID6	5	SD
ID7	2	
ID8	1	
ID9	1	PD
ID10	1	PD
ID11	3	PD
ID12	3	
ID13	3	PD
ID14	3	PD
ID15	2	PD
ID16	4	PD
ID17	2	PD
ID18	3	PD
ID19	2	PD
ID20	2	PD
ID21	3	PD
ID22	2	PD
ID23	3	PD

Abbreviations: BOR, best of response; PD, progressive disease; SD, stable disease.

**Table 3:** Treatment-related adverse events.

	No. (%)	
Adverse event	All grades	Grade 3 or 4
Anorexia	2 (9.0)	0
Fatigue	1 (4.5)	0
Edema	2 (9.0)	0
Abdominal pain	2 (9.0)	0
Pneumonia	1 (4.5)	1 (4.5)
Hyperbilirubinemia	2 (9.0)	2 (9.0)
Hypoalbuminemia	1 (4.5)	0
Hyponatremia	1 (4.5)	0
Anemia	2 (9.0)	0
Acute kidney injury	2 (9.0)	1 (4.5)
