# Supplementary material for: Safety and anti-tumor effects of vismodegib in patients with refractory advanced gastric cancer: A single-arm, phase-II trial
Source: J Cancer. 2022 Jan 9;13(4):1097–102. doi: 10.7150/jca.67050 (PMC8899372; doi:10.7150/jca.67050)
Supplement: Supplementary file 1 — Supplementary figures. [file jcav13p1097s1.pdf]

SUPPLEMENTARY FIGURE LEGENDS

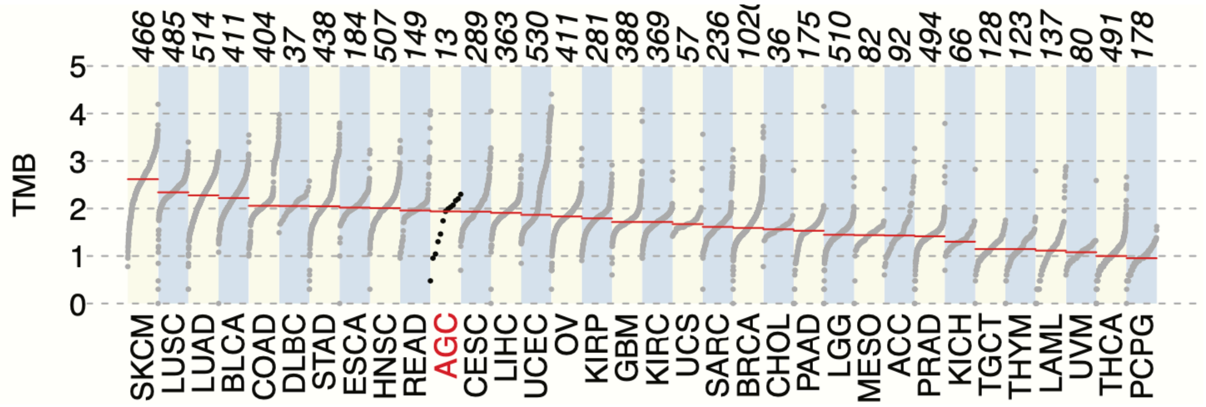

Figure S1. Mutational load of the advanced gastric cancer cohort.

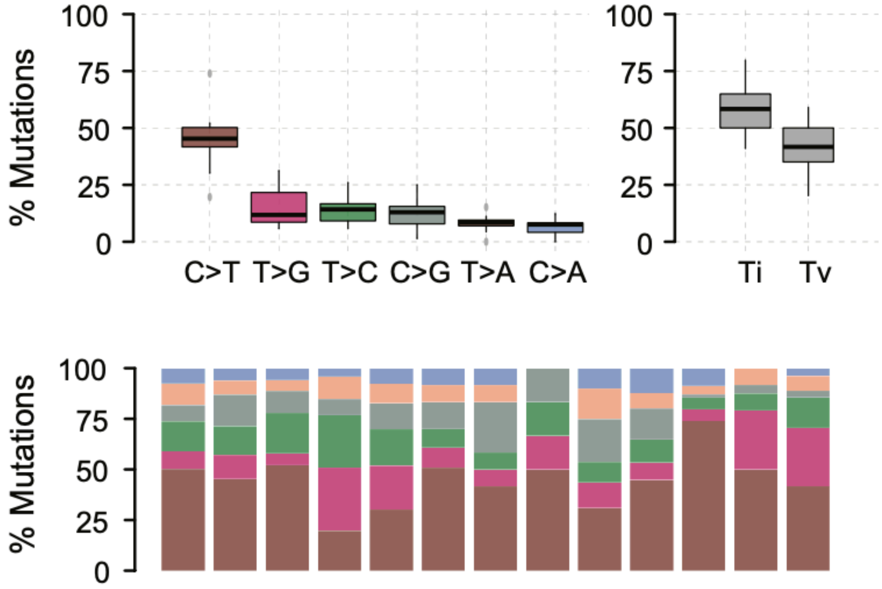

Figure S2. Classification of single-nucleotide variations.

SUPPLEMENTARY TABLES

Table S1. Focal copy number alterations identified by the Genomic Identification of Significant Targets in Cancer (GISTIC) algorithm in the study cohort.

Unique name	Number of genes	Number of samples	Variant classification	Cytoband	Wide peak limits	q-values
DP_5:16q11.2	11	8	Del	16q11.2	chr16:33294578-46723426	1.82E-06
DP_2:4q34.3	124	7	Del	4q34.3	chr4:160280551-191154276	0.0031908
AP_4:8q24.21	20	7	Amp	8q24.21	chr8:125730461-129029047	0.013246
DP_1:1p13.3	166	6	Del	1p13.3	chr1:102461462-121265007	0.084963
AP_2:7q31.2	11	5	Amp	7q31.2	chr7:115888553-116947663	0.09941
AP_1:7p11.2	11	6	Amp	7p11.2	chr7:53292358-56000794	0.14477
AP_3:8p23.1	2	6	Amp	8p23.1	chr8:12278702-12459239	0.14477
AP_5:12q14.3	39	5	Amp	12q14.3	chr12:65803552-71957037	0.14477
AP_7:17q12	7	4	Amp	17q12	chr17:37369541-37799779	0.14477
AP_8:19q12	18	5	Amp	19q12	chr19:23963713-31777953	0.14477
DP_3:8p21.2	18	3	Del	8p21.2	chr8:24209064-27325700	0.14822
DP_4:14q11.2	2	7	Del	14q11.2	chr14:19403660-19985576	0.14839
DP_6:21q22.3	11	8	Del	21q22.3	chr21:46924932-47715295	0.16068
AP_6:16q11.2	11	5	Amp	16q11.2	chr16:33783552-46733398	0.19368

Table S2. Genes contained in focal copy number alterations (CNA) in the study cohort: (A) amplification, (B) deletion.

(A)

cytoband	8q24.21	7q31.2	7p11.2	8p23.1	12q14.3	17q12	19q12	16q11.2
q value	0.013246	0.09941	0.14477	0.14477	0.14477	0.14477	0.14477	0.19368
residual q value	0.013246	0.09941	0.14477	0.14477	0.14477	0.14477	0.14477	0.19368
wide peak boundaries	chr8:125730461-129029047	chr7:115888553-116947663	chr7:53292358-56000794	chr8:12278702-12459239	chr12:65803552-71957037	chr17:37369541-37799779	chr19:23963713-31777953	chr16:33783552-46733398
genes in wide peak	hsa-mir-1206	CAPZA2	EGFR	FAM86B2	hsa-mir-1279	NEUROD2	CCNE1	hsa-mir-1826
	hsa-mir-1205	CAV1	HPVC1	LOC100506990	CPM	MED1	UQCRFS1	ORC6
	hsa-mir-1204	CAV2	SEC61G		IFNG	STARD3	URI1	VPS35
	MYC	MET	LANCL2		LYZ	CDK12	ZNF254	SHCBP1
	POU5F1B	WNT2	VOPP1		MDM2	PPP1R1B	ZNF536	ANKRD26P1
	PVT1	ST7	VSTM2A		CNOT2	FBXL20	POP4	LOC146481
	SQLE	TES	LOC285878		PTPRB	STAC2	TSHZ3	LOC283914
	MTSS1	ST7-AS1	14.Sep		PTPRR		PLEKHF1	FLJ26245
	KIAA0196	ST7-AS2	ZNF713		RAP1B		C19orf12	UBE2MP1
	TRIB1	ST7-OT3	FKBP9L		TSPAN8		DKFZp566F0947	LINC00273
	ZNF572	ST7-OT4	FLJ45974		YEATS4		LOC148145	LOC100130700
	LOC157381				HMGA2		LOC148189	
	FAM84B				DYRK2		LOC284395	
	NSMCE2				LGR5		VSTM2B	
	LOC727677				CCT2		RPSAP58	

LOC100130231	FRS2	ZNF726
MIR1205	CPSF6	LOC10010126<br>6
MIR1206	IRAK3	LOC10050583<br>5
MIR1204	GRIP1	
PCAT1	KCNMB4	
	IL22	
	TMBIM4	
	SLC35E3	
	IL26	
	CAND1	
	MDM1	
	NUP107	
	LLPH	
	HELB	
	RAB3IP	
	BEST3	
	RPSAP52	
	MSRB3	
	LRRC10	
	MIR1279	
	SNORA70G	
	MIR3913-2	
	MIR3913-1	
	LOC10050725<br>0	

(B)

cytoband	16q11.2	4q34.3	1p13.3	8p21.2	14q11.2	21q22.3
q value	1.82E-06	0.0031908	0.084963	0.14822	0.14839	0.16068
residual q value	1.82E-06	0.0031908	0.084963	0.14822	0.14894	0.16068
wide peak boundaries	chr16:33294578-46723426	chr4:160280551-191154276	chr1:102461462-121265007	chr8:24209064-27325700	chr14:19403660-19985576	chr21:46924932-47715295
genes in wide peak	hsa-mir-1826	hsa-mir-1305	hsa-mir-942	hsa-mir-548h-4	POTEG	COL6A1
	VPS35	hsa-mir-4276	hsa-mir-320b-1	ADRA1A	LOC642426	COL6A2
	SHCBP1	hsa-mir-548t	hsa-mir-4256	BNIP3L		LSS
	ANKRD26P1	hsa-mir-1979	hsa-mir-197	DPYSL2		SLC19A1
	LOC146481	AGA	ADORA3	PTK2B		MCM3AP
	LOC283914	SLC25A4	ALX3	GNRH1		FTCD
	LOC390705	CASP3	AMPD1	NEFM		PCBP3
	FLJ26245	CLCN3	AMPD2	NEFL		YBEY
	UBE2MP1	CPE	AMY1A	PPP2R2A		C21orf56
	LINC00273	DCTD	AMY1B	ADAM7		MCM3AP-AS1
	LOC100130700	F11	AMY1C	PNMA2		LOC100129027
		ACSL1	AMY2A	TRIM35		
		FAT1	AMY2B	ADAMDEC1		
		FRG1	RHOC	KCTD9		
		GK3P	ATP1A1	EBF2		
		GPM6A	ATP5F1	DOCK5		
		HMGB2	CAPZA1	STMN4		
		HPGD	CASQ2	CDCA2		

HSP90AA4P	CD2
ING2	CD53
IRF2	CD58
KLKB1	CHI3L2
MTNR1A	COL11A1
NEK1	CSF1
NPY1R	CELSR2
NPY5R	FCGR1B
MSMO1	GNAI3
TLL1	GNAT2
TLR3	GSTM1
VEGFC	GSTM2
GLRA3	GSTM3
SORBS2	GSTM4
SAP30	GSTM5
HAND2	HMGCS2
MFAP3L	HSD3B1
ADAM29	HSD3B2
ANXA10	IGSF3
KLHL2	KCNA2
SCRG1	KCNA3
DUX4	KCNA10
PALLD	KCNC4
ANP32C	KCND3
FAM149A	MOV10

FBXO8		NGF
DUX2		NHLH2
PDLIM3		NOTCH2
SPOCK3		NRAS
AADAT		OVGP1
GALNT7		PSMA5
CLDN22		PTGFRN
C4orf27		RAP1A
	1.Mar	SORT1
NEIL3		SARS
C4orf43		SLC16A1
UFSP2		STXBP3
DDX60		SYCP1
CDKN2AIP		TAF13
ODZ3		TBX15
LRP2BP		TSHB
FSTL5		WNT2B
STOX2		CSDE1
KIAA1430		TTF2
SH3RF1		SLC16A4
SPCS3		CD101
TRAPPC11		LRIG2
MLF1IP		TSPAN2
NBLA00301		BCAS2
WWC2		WARS2

CEP44	CEPT1
SNX25	VAV3
TKTL2	HBXIP
CBR4	AP4B1
MGC45800	PHTF1
DDX60L	AHCYL1
NAF1	WDR3
WDR17	MAN1A2
ZFP42	ADAM30
SPATA4	DDX20
ENPP6	NTNG1
ASB5	WDR47
C4orf38	CLCC1
C4orf39	PTPN22
TRIM60	PHGDH
TMEM192	CHIA
RWDD4	GPSM2
CCDC111	SLC25A24
TRIML2	HAO2
CCDC110	TRIM33
CYP4V2	RSBN1
LOC285441	GDAP2
LOC285501	FAM46C
LOC339975	ST7L
TRIML1	PRPF38B

ANKRD37	PRMT6
LOC389247	SLC22A15
TRIM61	RNPC3
HELT	LRIF1
LOC401164	CTTNBP2NL
FAM92A3	FAM212B
HSP90AA6P	TMEM167B
C4orf47	OLFML3
DUX4L4	AMIGO1
GALNTL6	KIAA1324
FRG2	RBM15
SLED1	DCLRE1B
FLJ38576	WDR77
DUX4L6	EPS8L3
DUX4L5	VTCN1
DUX4L3	DENND2D
MIR578	SIKE1
LINC00290	TRIM45
LOC728175	VANGL1
DUX4L2	GPR61
LOC731424	REG4
CLDN24	PROK1
LOC100288255	PSRC1
MIR1305	ATP1A1OS
MIR4276	FAM40A

MIR3945	ZNF697
LOC100505989	HENMT1
LOC100506013	MAB21L3
LOC100506085	ATXN7L2
LOC100506122	C1orf194
LOC100506229	HSD3BP4
	DRAM2
	C1orf88
	C1orf162
	SYT6
	NBPF4
	RP11-165H20.1
	DENND2C
	FNDC7
	UBL4B
	SPAG17
	HIPK1
	AKR7A2P1
	AKNAD1
	MAGI3
	FAM19A3
	FAM102B
	SYPL2
	CYB561D1
	PPM1J

	HIST2H2BA
	MYBPHL
	NBPF7
	SLC6A17
	MIR197
	LOC440600
	BCL2L15
	PGCP1
	SRG7
	CYMP
	LOC643441
	LOC644242
	LOC648740
	NBPF6
	FAM72B
	SCARNA2
	MIR942
	LOC100129138
	LOC100129269
	LOC100287722
	MIR320B1
	MIR4256
	LOC100506343
